# Structure-Activity Relationships of 3,3′-Phenylmethylene-bis-4-hydroxycoumarins: Selective and Potent Inhibitors of Gram-Positive Bacteria

**DOI:** 10.1155/2013/178649

**Published:** 2013-12-29

**Authors:** Kanokporn Petnapapun, Warinthorn Chavasiri, Pornthep Sompornpisut

**Affiliations:** Department of Chemistry, Faculty of Science, Chulalongkorn University, Bangkok 10330, Thailand

## Abstract

Dicoumarols and coumarin derivatives have shown a variety of pharmaceutical activities and have been found to be potent inhibitor for the NAD(P)H-dependent flavoproteins. In this report, dicoumarol and its derivatives containing the substituted benzene ring at the methylenebis position were synthesized and evaluated for their antibacterial activity against gram-positive bacteria: *Staphylococcus aureus* and *Bacillus subtilis*, and gram-negative bacteria: *Escherichia coli* and *Klebsiella* sp. The results showed that the synthesized dicoumarols affect cell growth but are selective against gram-positive over gram-negative bacterial cells. However, for most derivatives, the substitution of steric bulky benzene group on the methylenebis position appears to decrease in the efficacy of antibacterial effect. This finding is roughly described by the predicted poorer docked structure of the derivatives to a homology model of *S. aureus* flavoprotein. 3D-QSAR study highlighted structural features around the substituted benzene ring of dicoumarols as the antibacterial activity. CoMFA and CoMSIA contour maps support the idea that steric repulsion at the *para* position could diminish the antibacterial activity. The results of this study provide a better understanding of the molecular basis for the antibacterial activity of dicoumarols.

## 1. Introduction 

Dicoumarol (3,3′-methylene-bis-4-hydroxycoumarin) is a naturally coumarin-based compound which has long been used as an oral anticoagulant drug. It is metabolically produced from coumarin which was first isolated from both of the Tonka bean (*Dipteryx odorata*) and the sweet clover (*Melilotus alba and Melilotus officinalis*) [1]. It is now known to be present in many other plants. Dicoumarol derivative, warfarin (3-(*α*-acetonyl benzyl)-4-hydroxycoumarin), is commonly used as a natural anticoagulant for the prevention and treatment of excessive blood-clotting disorder [2]. Many dicoumarols and coumarin derivatives have also shown a variety of pharmaceutical activities such as antiinflammatory, antibacterial, antiviral, anticancer, anti-HIV, and antiproliferative properties [3–9]. Therefore, dicoumarols have received much attention for medical and pharmaceutical applications.

Dicoumarols and coumarin-based inhibitors exhibit a broad spectrum of activity against gram-positive bacteria [10, 11]. They have been shown to impede the growth of several bacteria strains, for instance, *Staphylococcus aureus*, *Bacillus anthracis, and Streptococcus pyogenes*. Another interesting property of dicoumarols lies in the effective anticancer activity [12]. Particularly, several evidences have shown that dicoumarol appears to be the most potent inhibitor which competes with NAD(P)H coenzyme for binding to the two-electron reduction quinone oxidoreductase [13], nitroreductase [14], azo-dyes azoreductase [15], and a ubiquitous flavoprotein found widely in various organisms. This flavoprotein is an antioxidant enzyme which plays a role in cellular protection by preventing the formation of free radical oxygen/nitrogen such as superoxide (O_2_^−^), hydroxyl radical (HO^*·*^), and nitric oxide (NO^*·*^). These reactive oxygen and nitrogen species (ROS and RNS) are toxic to cells. Inhibition of the redox flavoprotein by dicoumarol generates a high level of ROS and RNS which increase cellular toxicity and inhibit cell division. Thus, we hypothesize that the antiproliferative mechanism of action of dicoumarol might be via a major suppression of NAD(P)H-dependent flavoprotein activity. This hypothesis also relies on the available 3D crystal structures of dicoumarol-oxidoreductase complexes such as human NQO1 (pdb 2F1O), nitroreductase (pdb 1OOQ), and azoreductase (pdb 2Z9C) from *Escherichia coli*. From the crystallographic data, these proteins share great similarities in the secondary structure organization and the tertiary fold. They are homodimeric proteins with similar quaternary structure arrangement of the two monomers, providing two catalytic sites at the dimer interface. In each binding pocket, dicoumarol and flavin molecules interact with the surrounding residues of both subunits. Based on these structural studies, the results provided useful information about the potential and common target proteins for dicoumarol.

**Figure 1:**
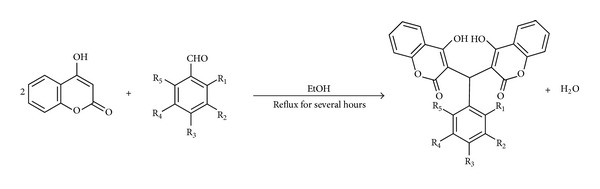
Synthesis of dicoumarol derivatives via condensation of 4-hydroxycoumarin with *ortho*-, *meta*-, and *para*-substituted benzaldehydes.

Due to the evolution of antibiotic resistance to existing drugs, the development of novel antibacterial drugs is essential. In search for more active compounds, it has been shown that the substitution of phenyl ring at the methylenebis position of dicoumarol exhibits interesting biological activity [16]. In this study, we focused on the synthesis and the antibacterial activity of a series of dicoumarol derivatives containing 3,3′-phenylmethylene-bis-4-hydroxycoumarins. Dicoumarols have been synthesized from the condensation reaction of 4-hydroxycoumarin with benzaldehydes (Figure 1). The synthesized compounds were tested for antibiotic susceptibility against gram-positive *S*. *aureus* and gram-negative *E. coli* bacteria through disk diffusion assay. The antibacterial activity tested against *E. coli* cells was used as a control. Since their antibacterial mode of action is still poorly understood, we made the use of existing protein sequences and structural information available in databases to identify the potential antibiotic target. A molecular modeling approach was employed to reveal the inhibition mechanism of the synthesized compounds on the NAD(P)H-dependent flavoprotein, a hypothetical protein target for dicoumarol. In the study, molecular docking was used to predict the binding mode of dicoumarol to the protein target and a 3D-QSAR approach was carried out for the analysis of structural features of dicoumarols as antibacterial activity. Understanding the molecular mechanism of drug action can provide the basis for the rational design of effective drugs.

## 2. Materials and Methods

### 2.1. Synthesis of Dicoumarols

All the chemicals and reagents were of analytical grade and were purchased from Sigma-Aldrich. All solvents used in the study were purified using standard methodology except for those which were reagent grade. The synthesis of dicoumarol and its derivatives was based on the previously described method [5]. Dicoumarol was synthesized by dissolving 4-hydroxycoumarin 1.00 g in 300 mL of boiling water; the solution was allowed to cool to 70°C and 10 mL of 40% aqueous formaldehyde was added with stirring. The mixture was then chilled. The crude product was then filtered off and washed well with water. The compound was obtained by recrystallization with ethanol.

A series of nineteen dicoumarols with various phenyl-based derivatives were synthesized by mixing 4-hydroxycoumarin with aromatic aldehydes containing different groups in *ortho, meta, or para* with the 2 : 1 ratio of molar equivalent (see Figures *S*1 and *S*2 in Supplementary Material available online at http://dx.doi.org/10.1155/2013/178649). The mixture was dissolved in ethanol. Each solution was refluxed until the solid began to precipitate. After cooling, the product was filtered and recrystallized with appropriate solvent. The purified compounds were identified by NMR. 1H NMR spectra were recorded on a Varian 400 MHz NMR spectrometer. The NMR samples were dissolved in deuterated chloroform (CDCl3) with tetramethylsilane (TMS) as an internal reference. The chemical shifts were assigned by a comparison with residue solvent protons.

### 2.2. Antibacterial Susceptibility Testing

The biological activity was undertaken by disk diffusion method [17]. The purified dicoumarol compounds were tested with gram-positive *Staphylococcus aureus* ATCC-6538 and *Bacillus subtilis* ATCC-6633, while gram-negative *Escherichia coli* ATCC-25922 and *Klebsiella *sp. TISTR-1843. A stock solution was prepared by dissolving 10 mg of the tested sample in 1000 *μ*L of DMSO solvents. A 20 *μ*L of each preparation was dropped into a disk of 6 mm in diameter. After incubation at 37°C for 24 hours, diameter of clear zone was measured. For positive control, penicillin G against *S. aureus* and *E. coli* and chloramphenicol against *B. subtilis* and *Klebsiella *sp. were used, while DMSO was used as negative control. The antibiotic susceptibility tests were undertaken twice.

### 2.3. Molecular Modeling 

#### 2.3.1. Comparative Modeling of a Protein Target from *S. aureus*

First, we used protein sequences from the three crystal structures human NQO1 (pdb 2F1O), nitroreductase (pdb 1OOQ), and azoreductase (pdb 2Z9C) from *E. coli* as primary information to identify homologous proteins from *S. aureus*. NCBI BLAST search for NAD(P)H-dependent flavodoxin-like proteins, a hypothetical target for dicoumarols, was performed through identified protein-encoding genes in the *S. aureus* genome. The resulting *S. aureus* flavoprotein that is showed to be the most similar to the reference protein sequences and complete sequence information was chosen as a putative dicoumarol-binding target protein for building a structure model. Based on the BLAST results, a comparative model of the *S. aureus* homodimeric protein was generated using the crystal structure of *E. coli* azoreductase as template. Sequence alignment and comparative modeling were carried out using Discovery Studio 2.5 (Accelrys Inc., San Diego, CA, USA). Subsequently, the best *S. aureus* protein structure with the lowest DOPE (the density optimisation potential energy) score was selected and subjected to structure validation using PROCHECK [18]. Then, energy minimization of the comparative protein structure was performed in the presence of dicoumarol and flavin mononucleotide (FMN) using AMBER 10 software package [19]. The structure of the protein-dicoumarol-FMN complex was prepared by superimposing the model onto the template structure. The force field parameters of FMN were taken from Lugsanangarm et al. [20], while those of dicoumarol were developed using the same protocol as described in the literature [20].

#### 2.3.2. Molecular Docking

To evaluate the binding mode of dicoumarol with the surrounding residues of the *S. aureus* flavoprotein, the flexible docking was performed using AutoDock Vina [21]. 3D structures of all synthesized dicoumarol derivatives were built and optimized at the semiempirical AM1 level using the Gaussian 03 program [22]. For the target protein, we removed the dicoumarol molecule and all the hydrogen atoms of the energy-minimized comparative model, while the FMN molecule remained in the binding pocket of the receptor during the docking simulation. Gasteiger partial charges, atom types, and polar hydrogen were assigned to the protein and all ligands using AutoDock Tools (ADT) [23]. The torsional bonds of ligands were set free. The center of the 40 × 40 × 40 Å grid box was estimated from the dicoumarol position present in the crystal structure. The exhaustiveness parameter was set to 64. Top ten docked conformations from each calculation were ranked according to the binding energy and clustered on the basis of root-mean-square deviation (RMSD) of the ligand atoms. For each compound, the docked poses that adopt an orientation similar to that of the experimental X-ray structure (RMSD <3Å) were chosen as a representative bound conformation. Structure analysis and visualization were performed using VMD software [24].

#### 2.3.3. D-QSAR Models

All the synthesized dicoumarol derivatives and their inhibition zone were used to construct CoMFA and CoMSIA models using the program SYBYL (Tripos International, Missouri, USA). The training set of 14 dicoumarol derivatives was selected on the basis of structural diversity and wide range of activities and the test set contains 5 compounds (Table 1). Model building of the compounds was already described in the previous section (molecular docking). Atomic charges of the compounds were assigned according to Gasteiger-Huckel method [25]. Structural alignment of the compounds was performed using the SYBYL automatic alignment feature. For CoMFA, steric and electrostatic fields of the compounds were automatically computed using the standard CoMFA method with default parameters. Analogously, the calculated CoMSIA fields are associated with five descriptors including steric, electrostatic, hydrophobic, hydrogen bond donor, and hydrogen bond acceptor. The partial least squares (PLS) analysis was carried out using the “leave-one-out” cross-validation protocol to choose the optimum number of components (ONC) that does not exceed one-third of the number of the training compounds but yields the highest cross-validated correlation coefficient (*q* ^2^). Subsequently, non-cross-validation analysis was performed with the same ONC to calculate the conventional correlation coefficient (*r* ^2^), standard error of estimation (SEE), and *F* values.

## 3. Results and Discussion

### 3.1. Synthesis of Dicoumarols

Dicoumarol and nineteen dicoumarol derivatives were synthesized from the condensation reaction between two-mole equivalent of 4-hydroxycoumarin and one-mole equivalent of corresponding aldehydes. In preparation of dicoumarol, formaldehyde rapidly reacted with 4-hydroxycoumarin in hot water. The compound derivatives were from the condensation of 4-hydroxycoumarin with different substituted aromatic aldehydes in ethanol for 24 hours. This method is useful for aromatic aldehydes because the insoluble products were easily separated by filtration.

The synthesized compounds have an excellent yield. The yield of all dicoumarol derivatives was found to be in the range of 70–90% (Table 1). Most of dicoumarols were soluble in CDCl_3_; DMSO was used as a solvent for dicoumarols which were insoluble in CDCl_3_. For dicoumarol moiety, 2H integration of H-5 was shown as doublet around 7.80–8.10 ppm and 2H integration of H-6 was seen as triplet at 7.50–7.60 ppm. The overlapped 4H integration of H-7 and H-8 at 7.20–7.45 ppm was detected. The typical 1H integration of CH methylene bridge was observed in a range between 3.85 and 6.70 ppm depending on the R substitution at the bridge carbon. Characteristic spectroscopic data of the synthesized compounds are provided in the supplementary data.

### 3.2. Antibacterial Susceptibility Testing

Two gram-positive bacteria, *S. aureus,* and *B. subtilis,* and two gram-negative bacteria, *E. coli *and *Klebsiella *sp., were selected for the assay of cell-growth inhibition. Data of antibacterial effects of tested compounds are presented in Table 1. The results showed that all the synthesized compounds had no inhibitory activity against *E. coli* and *Klebsiella *sp. strains whereas their antibacterial effects were found for *S. aureus* and *B. subtilis*. The antibacterial activities of the dicoumarol derivatives against *S. aureus* are in a range of the clear zone from 16 to 26 mm. Although the antibacterial susceptibility was not great when compared to penicillin G (inhibition zone = 40 mm), the halide-substituted benzene ring of the derivatives (CID 2, 3, 4, 5, and 6) had comparable activity to the parent dicoumarol (CID 1). The derivative CID 16 had the lowest inhibitory activity. For *B. subtilis*, the results show that CID 13 exhibits the greatest antibacterial activity with an average clear zone of 38 mm. Again the derivatives containing halide group showed the activity comparable to the parent dicoumarol, especially CID 2 exhibiting the equivalent zone of the inhibition (32 mm). The tertiary butyl group substituent at *para* position (CID 10) showed the lowest activity that is 20 mm. It appears that the antibacterial effect of the dicoumarol derivatives against *B. subtilis* and *S. aureus* has a similar trend. The steric or bulky hydrophobic properties at the *para* position tend to decrease the inhibition zone of dicoumarol, whilst the halide-substituted benzene ring of the derivatives (CID 2, 3, 4, 5, and 6) tends to increase the activity.

The results are consistent with the previously observed inhibition of dicoumarols that act selectively against gram-positive microorganisms [10, 26]. One of the possible explanations is due to a permeability barrier to dicoumarol molecule because structure and properties of cell membranes between the two bacterial cells are different. The presence of outer membrane of peptidoglycan in *E. coli* may prevent the transport of dicoumarols into the cell. There is yet no evidence for the relation between molecular properties of the tested compounds and their transport across the membrane. Our results seem to be likely the case, but the study is currently limited to test such hypothesis.

Among the active compounds, the results show that the parent dicoumarol exhibits the high antibacterial activity with an average clear zone of 28 mm for *S. aureus* and of 32 mm for *B. subtilis*. In case of *S. aureus*, addition of the benzene ring at the methylene bridge position does not increase the activity. Analysis of substitution effect on the antibacterial activity against *S. aureus* revealed that the compounds containing halide and nitro groups exhibited the inhibition zone in a range of 24–26 mm, whereas those containing the methyl, methoxy, and hydroxyl groups had a narrower clear zone of 18–23 mm. This indicated that the presence of electron withdrawing group in benzene ring gave a more potent inhibitor compared to the substituted electron donating groups. Particularly, the antibacterial activity of the compound with three methoxy substitutions (CID 16) was the lowest among the active compounds. A fairly similar substitution effect on the activity against *B. subtilis* was observed, except for CID 13 (38 mm) and 19 (31 mm). Nevertheless, from the results, adding steric or bulky hydrophobic properties at the *para* position tends to diminish the antibacterial inhibition zone of both *S. aureus* and *B. subtilis* cells.

### 3.3. Exploring Putative Target and Binding Site for Dicoumarols

Next, we attempted to explain the observed decrease in the efficacy of derivatives with steric bulk at the *para *position of the phenyl ring in terms of the structure property and the binding of the dicoumarols. Initially, we looked for the potential dicoumarol target in DrugBank (http://www.drugbank.ca), a database containing some information on drug targets. In search for potential targets of dicoumarols, we found vitamin K epoxide reductase complex subunit 1, NAD(P)H dehydrogenase [quinone], quinone oxidoreductase, and cytochrome P450 2C9. Among these proteins, the 3D crystal structure is available only for a class of quinone or NAD(P)H-dependent oxidoreductase in complex with dicoumarol.

A further analysis of the NAD(P)H-dependent oxidoreductase proteins from *S. aureus* genome through NCBI database identified potential antibacterial targets including quinone reductase, nitroreductase, flavin reductase, and FMN oxidoreductase. The hit proteins from the search were subsequently screened to identify the protein target, of which the 3D structure is available as a template. It appears that an FMN-dependent NADH-azoreductase had similar sizes and homology suited for further investigation through a molecular modeling approach.

**Figure 2:**
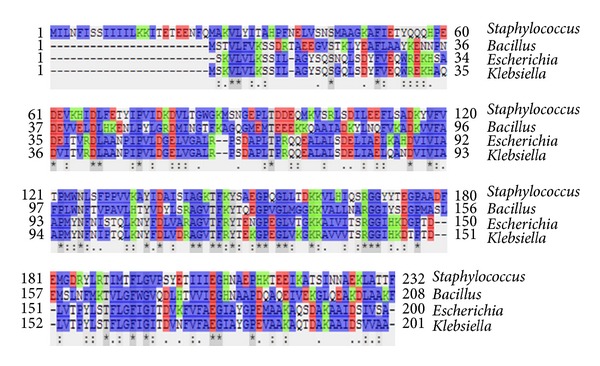
Multiple sequence alignment of azoreductase from *Staphylococcus aureus*, *Bacillus subtilis*, *Escherichia coli,* and *Klebsiella *sp. Amino acid residues are colored to indicate their similarity: blue = hydrophobic, red = acidic, and green = basic residues. For comparative model, the sequence identity with the template is of 34%.

**Figure 3:**
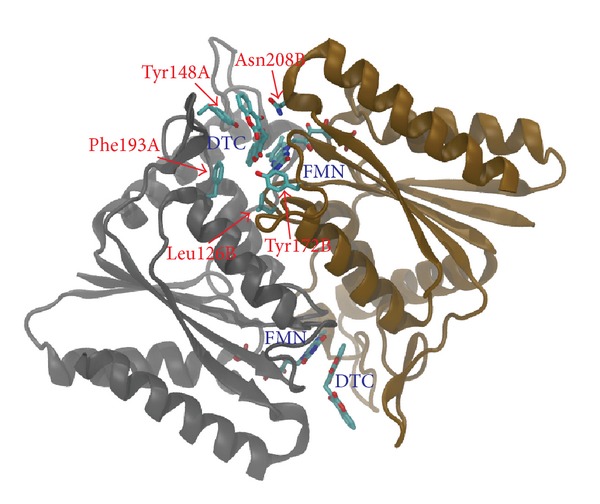
Comparative model of *S. aureus* azoreductase in complex with FMN and dicoumarol. The protein model is a homodimer composed of two ligand-binding sites located at the dimer interface. FMN, dicoumarol (DTC), and the conserved binding residues are shown in stick representation.

A comparative model of NAD(P)H-dependent flavoprotein from *S. aureus* (A9635) was constructed based on the 3D structure of dicoumarol-binding azoreductase (2Z9C) with 34% of sequence identity (Figure 2). The model is a homodimer of ternary complex containing the enzyme, FMN, and dicoumarol (Figure 3). Each monomer contains 206 out of 232 residues of the full-length protein. Dicoumarol is located at the dimer interface and interacts with active site residues of both chains.

**Figure 4:**
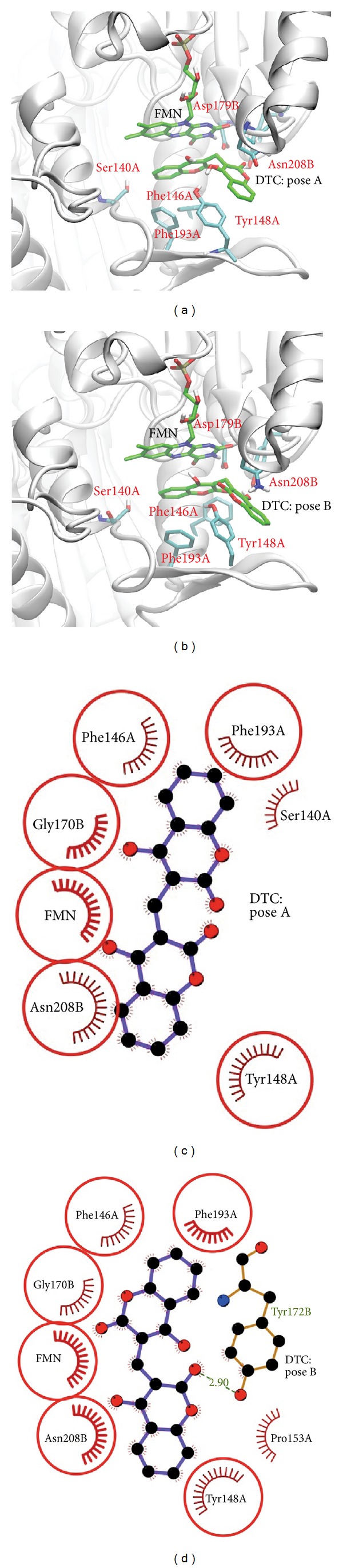
Docked orientations and interactions of dicoumarol in the enzyme binding sites. (a) and (b) compare the ligand poses A and poses B, respectively. The surrounding conserved residues, FMN, and dicoumarol molecules are in stick representation. (c) and (d) are schematic 2D diagrams of protein-ligand binding poses A and B, respectively. Spoked arcs represent residues making hydrophobic contacts with the ligand, whereas dashed lines represent hydrogen bonds between the atoms involved. The 2D representation was created using the program LigPlot [27].

To further validate the quality of the homology model, dicoumarol was docked into the enzyme binding pocket. The results show that one of the ten energetically most favorable docking poses adopted ligand orientation similar to that of the comparative model of the ternary complex but differed slightly in the depth of the ligand position within the binding pocket (Figure 4(a)). The docking model revealed sandwich-stacking interactions of dicoumarol with the FMN cofactor and the residues Phe146A, Tyr148A, Phe193A, Gly170B, and Asn208B (“A” and “B” indicate the protein chain) which are mainly hydrophobic (Figure 4(c)). Particularly, one of coumarin rings stacks between the isoalloxazine ring of FMN and the sidechain of Tyr148A. The pi-pi stacking motif is similar to the binding mode of conserved residues found in the X-ray structures of human NQO1 quinone reductase and *E. coli* azoreductase. Thus, the conserved binding mode suggested that the *S. aureus* azoreductase might be a potential protein target for antiproliferative activity of dicoumarols.

Further inspection of docking results illustrated an interesting binding mode of dicoumarols. Given the structure of the binding site, the two coumarin rings possibly adopted two alternative orientations, denoted as pose A and pose B. As described in the previous paragraph, the ligand orientation of pose A is similar to that of the crystal structures. Pose B shows a very similar binding pose in a way that one of the two coumarin rings flips approximately 180 degrees with respect to pose A (Figure 4). The binding mode of pose B revealed a similar pi-pi stacking motif, with additional hydrogen bonding between the hydroxyl hydrogen of the flipped coumarin ring and Tyr172B sidechain. Considering molecular shape of the ligands, the two binding poses are similar. Ring flipping of the ligands has no obvious effect on the ring stacking interactions. This can be seen as the binding energies between the two poses are not significantly different (Table 2). At this point, we cannot distinguish between these possibilities; however, the previous study by Nolan et al. also observed similar docked poses of dicoumarols in the active site of human NQO1 [16].

**Figure 5:**
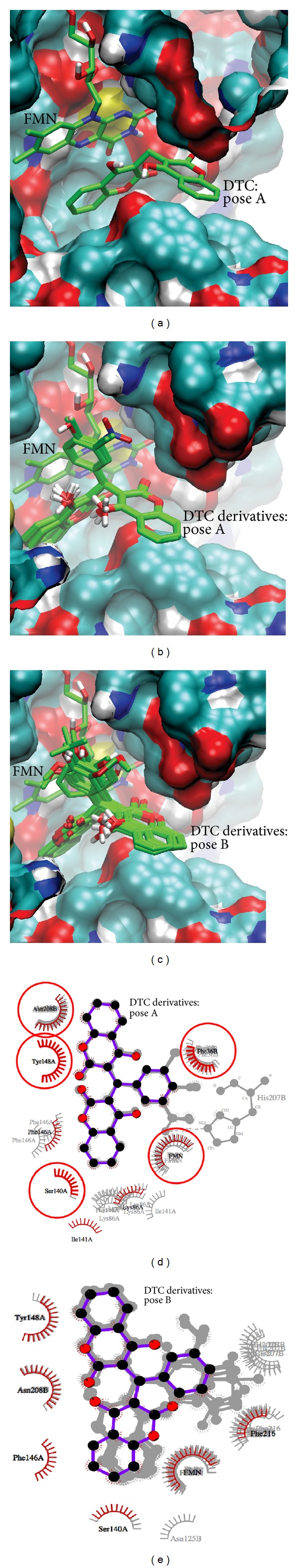
Docked orientations and interactions of dicoumarol derivatives in the enzyme binding site. (b) and (c) are the docking poses A and B of compound derivatives in comparison with pose A of dicoumarol (a) in the pocket (surface representation). (c) and (d) are schematic 2D diagrams of protein-ligand binding poses A and B, respectively.

The docking studies of 19 dicoumarol derivatives suggested that the *S. aureus* azoreductase model possesses a large binding cavity and can accommodate larger ligands. For all docked conformations, these compounds possess a similar orientation of coumarin rings with RMSD <2Å (Figure 5). Most compounds bind in the binding pocket with a more shallow position than that previously observed for the docked poses of the parent dicoumarol. A shift in position of the derivatives may be due to steric hindrance of the bulky benzene ring. By comparing the resulting ligand poses, the majority of the binding modes of all derivatives have fallen into two binding poses A and B similar to docking results of the parent compound. Both binding poses share common interactions as the coumarin ring forms pi-pi stacking with the FMN cofactor and Tyr148A. Inspection of these two ligand-receptor complexes identified several contact residues including Lys86A, Ser140A, Ile141A, Phe146A, Tyr148A, Phe36B, His206B, and Asn208B. It should be noted that the interactions of these residues are predominantly hydrophobic.

Analysis of docking results suggests that the parent compound produced, on average, good binding energy when compared to its derivatives, except for CID 4, 11, and 20 (Table 2). The good agreement between the experimental activity and the docking supports the idea that adding a benzene ring at the methylenebis position could reduce antibacterial activity. However, no clear correlation could be established between the calculated binding energy and experimentally determined inhibition zone for the tested compounds.

It should be noted that the relation between the experimental work and molecular docking is speculative. There is no direct evidence that the derivatives indeed inhibit or are binding to the target enzyme. The results could be greatly strengthened if one can experimentally prove that this is truly the case. At the time being, we have limited resources for the validation.

### 3.4. Analysis of 3D-QSAR Results

In an attempt to establish a relationship between the structure and activities, we performed CoMFA and CoMSIA analysis using the inhibition zone of *S. aureus*. Statistical results of CoMFA and CoMSIA studies are summarized in Table 3. For the CoMFA model, PLS analysis showed a *q* ^2^ value of 0.694 with 2 components, *r* ^2^ value of 0.894 with SEE of 0.929, and *F* value of 46.3. The steric and electrostatic contributions were 59.2% and 40.8%, respectively. This indicated that the steric properties of the substituents have more impact on the antibacterial activity than the electrostatic properties.

The PLS results of CoMSIA analysis showed a *q* ^2^ of 0.683 with an optimized component number of 4. The non-cross-validation analysis of CoMSIA models gave a high *r* ^2^ value of 0.977 with a SEE of 0.479 and *F* value of 94.8. The predominant field contributions from CoMSIA were electrostatic (25.6%) and hydrogen bond donor (31.7%), implying the importance of the electrostatic and hydrogen bond donor of the substituent for the activity. The other contributions, steric (10.3%), hydrophobic (19.9%), and the hydrogen bond acceptor (9.5%), gave minor effects.

**Figure 6:**
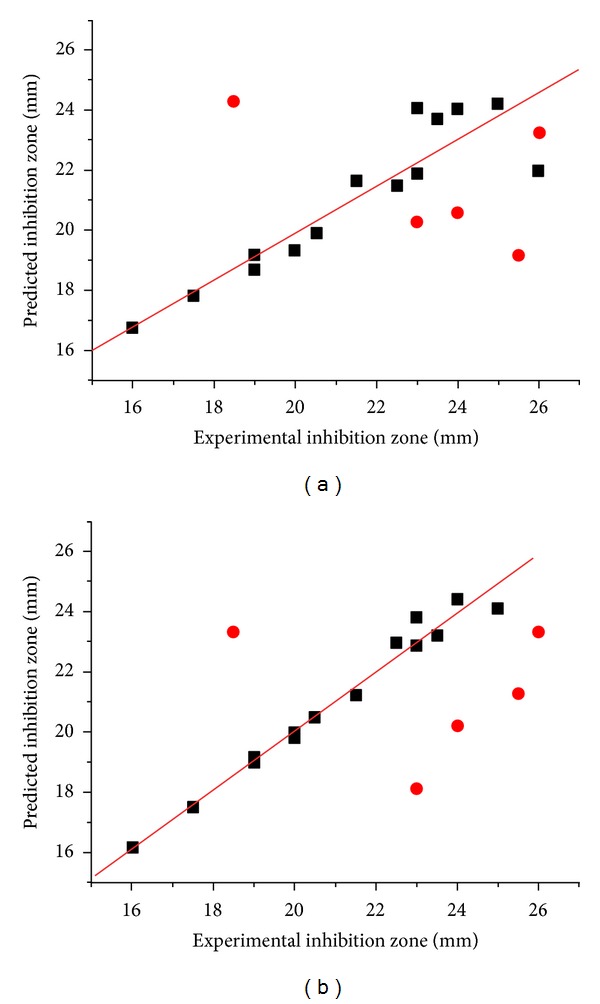
Plots between the experimental and predicted biological activities of training (filled square) and test set (filled circle): (a) CoMFA plot and (b) CoMSIA plot.

The predicted inhibition zone versus their experimental data of training set and test set compounds are illustrated in Figure 6. Although both models gave a fairly good quality of the activity prediction with *q* ^2^ > 0.6 for the training set, the predictive quality of the test set is not great. An average deviation between the predicted and experimental inhibition zone was about 4 mm (Table 4). Particularly, the activities of the two CID, 2 and 18, were poorly predicted. Compound 2 has the highest value in the set, whereas compound 18 exhibits almost the lowest activity.

**Figure 7:**
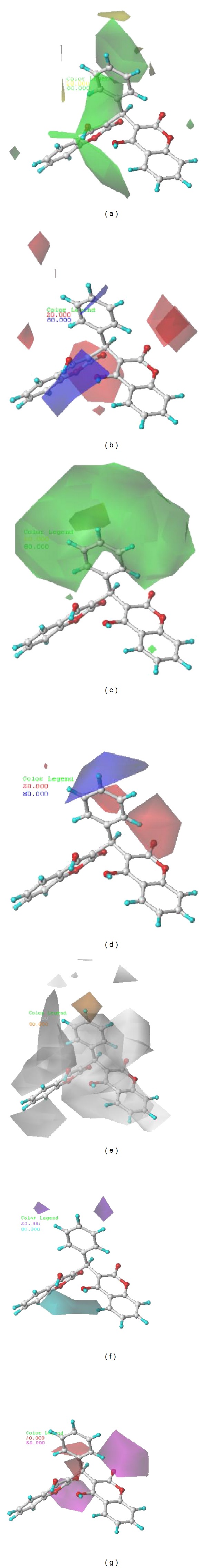
The stdev*coeff. contour maps. (a) and (b) represent CoMFA steric and electrostatic, respectively. (c) to (f) represent CoMSIA steric, electrostatic, hydrophobic, hydrogen bond donor, and hydrogen bond acceptor, respectively. Steric field is represented by green and yellow contour maps. Electrostatic field is represented by blue and red contour maps. Hydrophobic field is represented by orange and white contour maps. Hydrogen bond donor field is represented by cyan and purple contour maps. Hydrogen bond acceptor field is represented by magenta and red contour maps. All the contours represented the default 80% and 20% level contributions for favorable and unfavorable regions, respectively.

The effects of field contributions are illustrated by CoMFA and CoMSIA contour maps (Figures 7(a)-7(b)). Our interpretation is focused on the effects of substituted phenyl ring to the antibacterial activity. For CoMFA, a green contour was found near the plane of substituted phenyl ring of compound, while a yellow contour was located in the vicinity of the R3 substituent. This indicates that the bulky substituents at the *para* position were unfavorable and might have negative effect on the activity of the compounds. Therefore, the activity of the CID 10, 16, 17, 18, and 20 is relatively low. For electrostatic maps, a red contour was found around the R1-position. This corresponds well with the –OMe substitution at *ortho* position of CID 19 and 20 with an electron-donating group and hence these two compounds exhibit moderate and low activities, respectively.

For CoMSIA contour maps, steric and electrostatic are similar to those of CoMFA as shown by a yellow contour at the *para* position (inside a big green contour) and a red contour at the R1-position (Figures 7(c)-7(d)). Hydrophobic contour map from CoMSIA is shown in Figure 7(e). An orange contour found at the R3-position suggests that a hydrophobic substituent at this site is expected to improve the activity. For hydrogen bond donor contour maps (Figure 7(f)), small purple contour located near the R2 (or R4) substituent indicated that the position requires the hydrogen bond acceptor substituent. A magenta contour for hydrogen bond acceptor group suggested that the R1 substituent is in favor of hydrogen bond acceptor (Figure 7(g)).

## 4. Conclusion

We describe the synthesis of several 3,3′-phenylmethylene-bis-4-hydroxycoumarins containing *ortho, meta*, and *para *substitutions on the benzene ring and their antibacterial activity against *S. aureus*, *B. subtilis*, *E. coli,* and *Klebsiella *sp. The selectivity of these compounds for gram-positive bacteria is clearly demonstrated. The compounds are reasonable, although not great, inhibitors of the growth of the gram-positive bacteria compared to the parent dicoumarol compound. For most compounds, the substitutions of bulky benzene group on the methylenebis position do not significantly increase in inhibitory activity. This finding is broadly explained by the predicted poorer docked structure of the derivatives to a homology model of *S. aureus* flavoprotein. The QSAR results illustrate a relationship between the antibacterial activity and dicoumarol derivatives. Both CoMFA and CoMSIA models provided chemical and structural information insight into the effects of steric, electrostatic, hydrophobic, hydrogen-bond donor, and acceptor fields around the substituted benzene ring of dicoumarol on biological activity. The findings of the study provide a better understanding of the inhibition mechanism of dicoumarols and may be useful guiding structural design of more effective antibacterial agents.

## Supplementary Material

All spectroscopic evidences and methodology for synthesis of twenty compounds were shown in this supplementary material. The 1H NMR spectra of the twenty synthesized compounds were obtained on a Varian spectrometer that was operated at 400 MHz of proton frequency. The NMR samples were dissolved in CDCl_3_ with tetramethylsilane (TMS) as an internal reference or DMSO-d_6_. For compound 1 was synthesized by dissolving 4-hydroxycoumarin 1.00 g (2.97 mmol) in 300 mL of boiling water, the solution was allowed to cool to 70°C and 10 mL of 40% aqueous formaldehyde was added with stirring. The mixture was then chilled, the crude product was filtered off and washed well with water, dried and recrystallized with EtOH.
A series of nineteen dicoumarols (compound **2-20**) with various phenyl derivatives were synthesized by mixing 4-hydroxycoumarin (15 mmol) and selected aromatic aldehydes (7.5 mmol) with different substituents at *ortho*, *meta* or *para* (2:1 ratio of molar equivalent). The mixture was dissolved in EtOH 45 mL. Each solution was refluxed approximately 24 hours until the solid began to precipitate. After cooling, the product was filtered and recrystallized with EtOH. The purified compounds were identified by NMR spectrometer.

## Figures and Tables

**Table 1:** Reaction yield and antibacterial properties of dicoumarols.

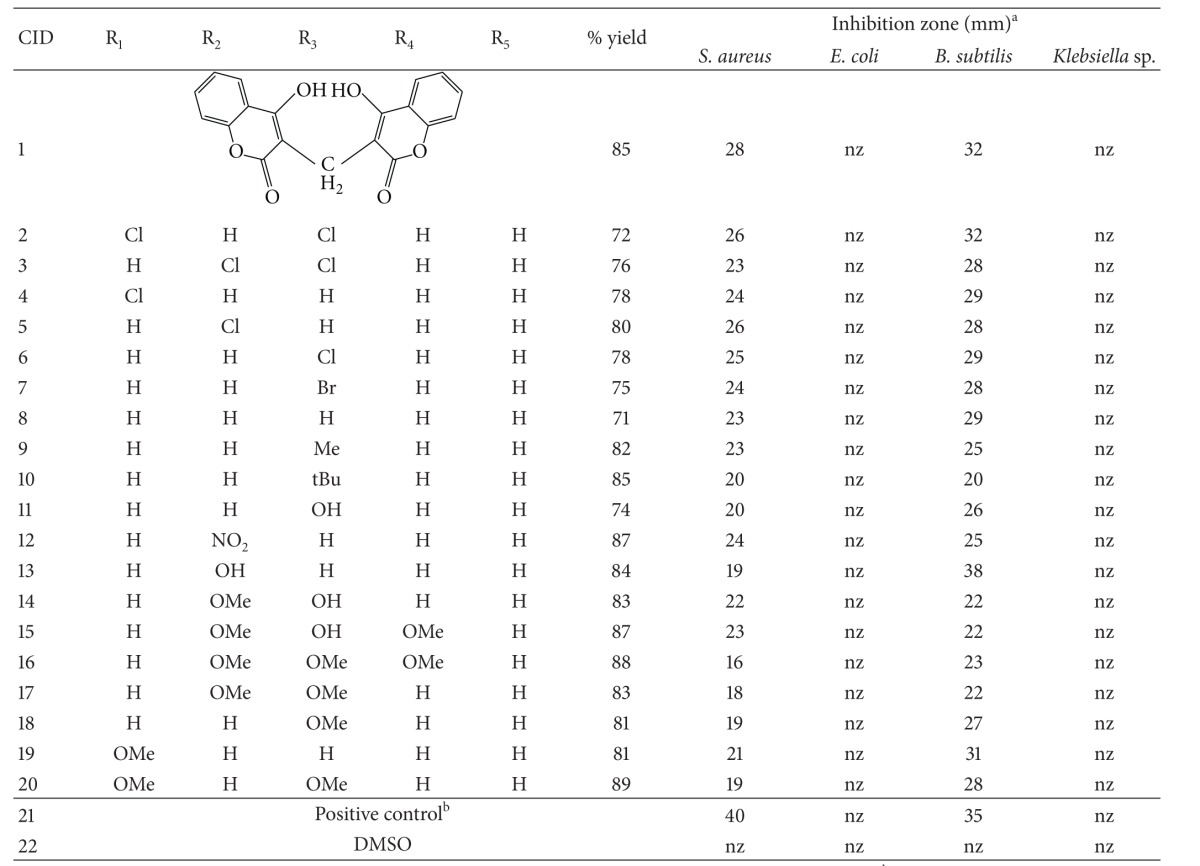

Note: ^a^the antibiotic tests were undertaken twice. Average values are reported. nz: no inhibition zone; nf: not found. ^b^Positive control is penicillin G for *S. aureus* and *E. coli*, while chloramphenicol was used for *B.  subtilis* and *Klebsiella *sp.

**Table 2:** Binding energy of dicoumarols.

CID	Binding energy (kcal/mole)	
	Pose A	Pose B
1	−9.3	−9.3
2	nf	−8.9
3	−9.0	−8.8
4	−9.4	−9.6
5	−9.0	−8.7
6	−8.9	nf
7	−8.5	−8.5
8	−8.8	−8.8
9	−8.1	−9.5
10	−8.4	−8.5
11	−9.6	−9.3
12	−9.0	−8.8
13	nf	−8.6
14	−8.2	−8.2
15	−8.6	−8.5
16	nf	−8.4
17	nf	−8.5
18	−8.3	nf
19	−8.2	nf
20	−9.4	−9.6

Note: nf: not found.

**Table 3:** Statistical results of CoMFA and CoMSIA models.

Models	Descriptors	Statistical parameters					
		*q* ^2^	*r* ^2^	*N *	SEE	*F *	Field contribution (%)
CoMFA	S/E	0.694	0.894	2	0.929	46.3	59.2/40.8
CoMSIA	S/E/H/D/A	0.683	0.977	4	0.479	94.8	10.3/25.6/19.9/31.7/9.5

S: steric field; E: electrostatic field; H: hydrophobic field; D: donor; A: acceptor; *q* ^2^: cross-validated correlation coefficient; *r* ^2^: non-cross-validated correlation coefficient; *N*: optimal number of components; SEE: standard error of estimate; *F*: *F* test.

**Table 4:** Experimental and predicted activities of test set compounds.

CID	Inhibition zone (mm)	CoMFA		CoMSIA	
		Predicted (mm)	Residual (mm)	Predicted (mm)	Residual (mm)
2	26	19	7	21	5
4	24	21	3	20	4
5	26	23	3	23	3
15	23	20	3	18	5
18	19	24	5	23	4

		Avg	4	Avg	4

## Abbreviations

NAD(P)H: The reduced form of nicotinamide adenine dinucleotide (phosphate)
PDB: Protein data bank
FMN: Flavin mononucleotide
NQO1: Quinone oxidoreductase 1
QSAR: Quantitative structure-activity relationship
NCBI: National Center for Biotechnology Information
BLAST: Basic local alignment search tool
DMSO: Dimethyl sulfoxide
CoMFA: Comparative molecular field analysis
CoMSIA: Comparative molecular similarity indices analysis.
