# Supplementary material for: Structure-Activity Relationships of 3,3′-Phenylmethylene-bis-4-hydroxycoumarins: Selective and Potent Inhibitors of Gram-Positive Bacteria
Source: ScientificWorldJournal. 2013 Dec 29;2013:178649. doi: 10.1155/2013/178649 (PMC3891237; doi:10.1155/2013/178649)
Supplement: Supplementary file 1 — All spectroscopic evidences and methodology for synthesis of twenty compounds were shown in this supplementary material. The 1H NMR spectra of the twenty synthesized compounds were obtained on a Varian spectrometer that was operated at 400 MHz of proton frequency. The NMR samples were dissolved in CDCl3 with tetramethylsilane (TMS) as an internal reference or DMSO-d6. For compound 1 was synthesized by dissolving 4-hydroxycoumarin 1.00 g (2.97 mmol) in 300 mL of boiling water, the solution was allowed to cool to 70°C and 10 mL of 40% aqueous formaldehyde was added with stirring. The mixture was then chilled, the crude product was filtered off and washed well with water, dried and recrystallized with EtOH. A series of nineteen dicoumarols (compound 2-20) with various phenyl derivatives were synthesized by mixing 4-hydroxycoumarin (15 mmol) and selected aromatic aldehydes (7.5 mmol) with different substituents at ortho, meta or para (2:1 ratio of molar equivalent). The mixture was dissolved in EtOH 45 mL. Each solution was refluxed approximately 24 hours until the solid began to precipitate. After cooling, the product was filtered and recrystallized with EtOH. The purified compounds were identified by NMR spectrometer. [file 178649.f1.pdf]

Structure-activity relationships of 3,3'-phenylmethylene-bis-4-hydroxycoumarins: selective and potent inhibitors of gram-positive bacteria

Kanokporn Petnapapun, Warinthorn Chavasiri and Pornthep Sompornpisut*

Supplementary information

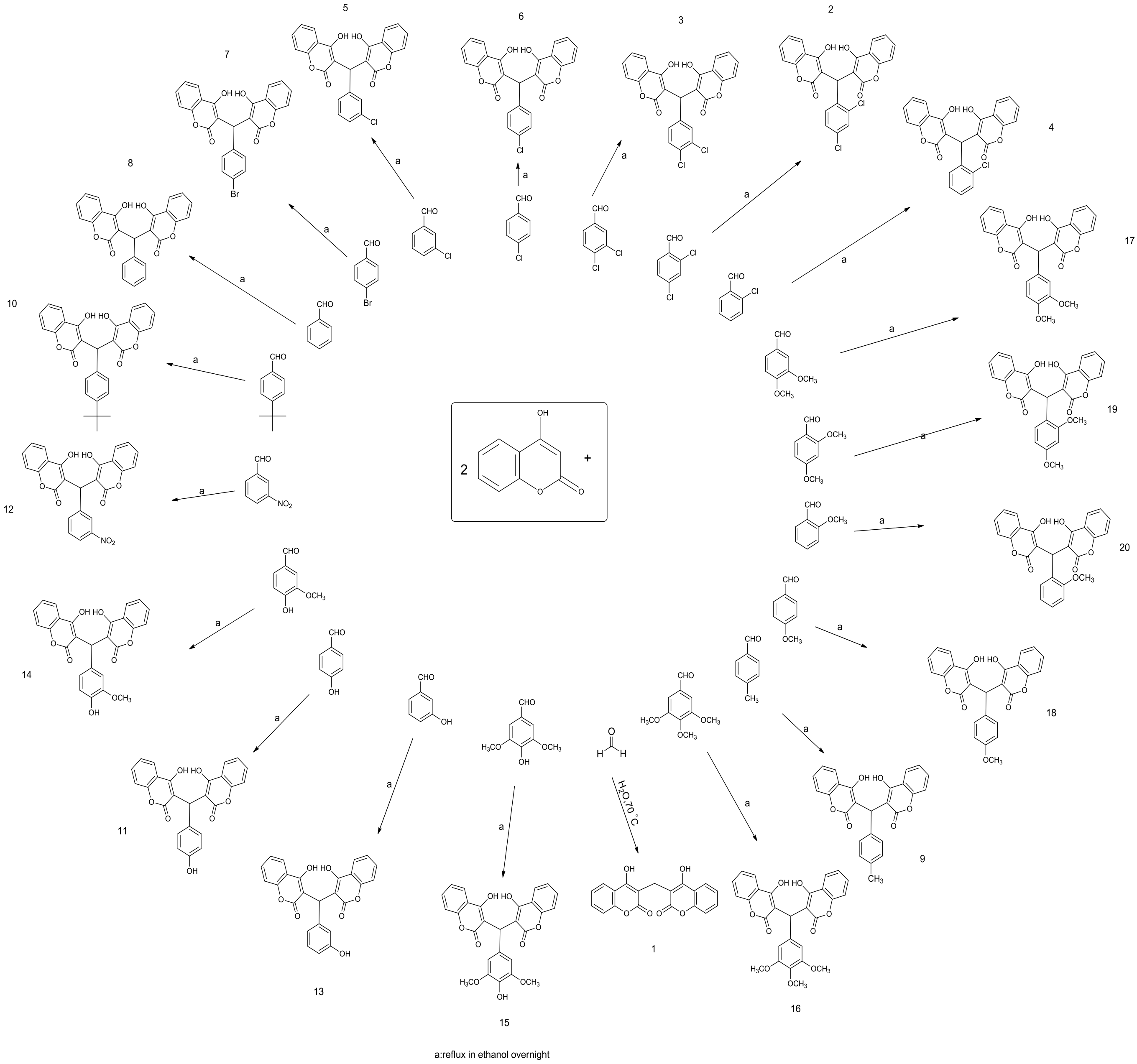

Figure S1 Synthesis of dicoumarol derivatives via condensation of 4-hydroxycoumarin with ortho-, meta- and para-substituted benzaldehydes

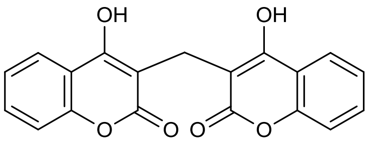

1

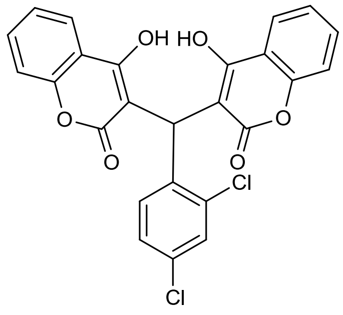

2

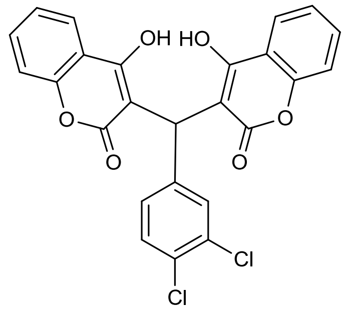

3

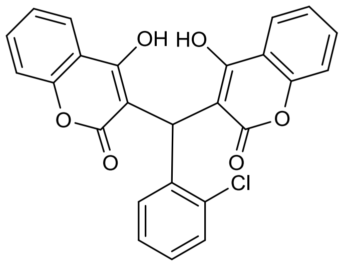

4

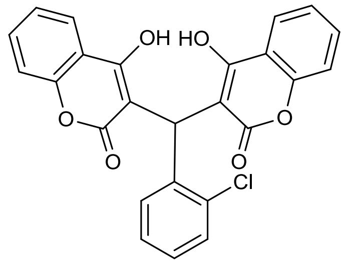

5

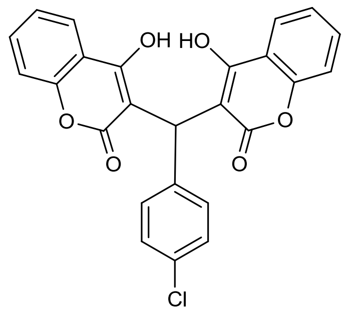

6

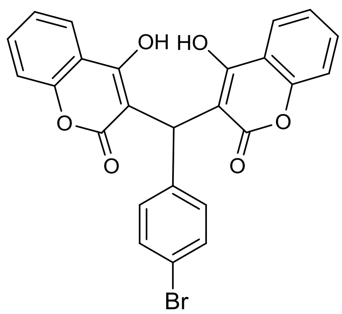

7

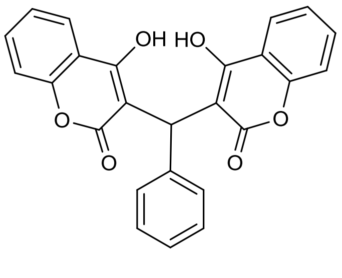

8

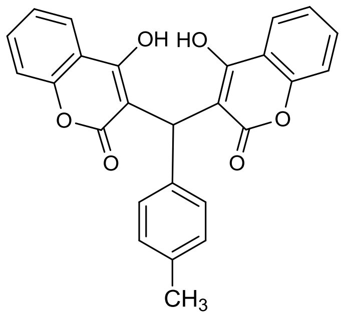

9

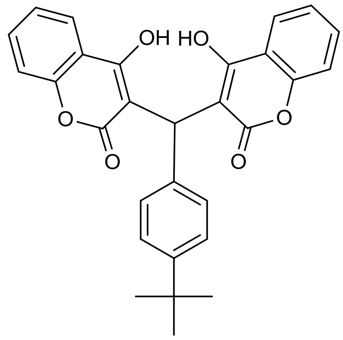

10

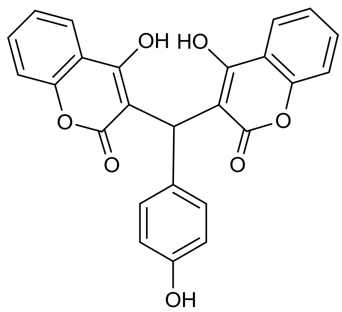

11

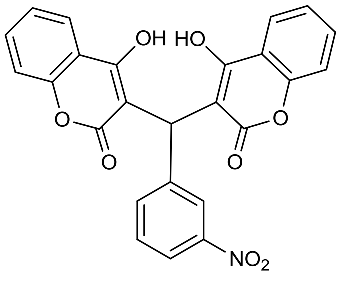

12

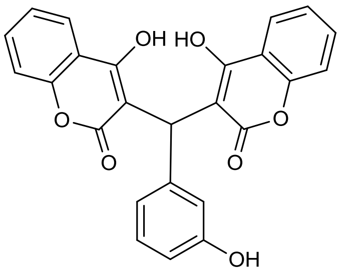

13

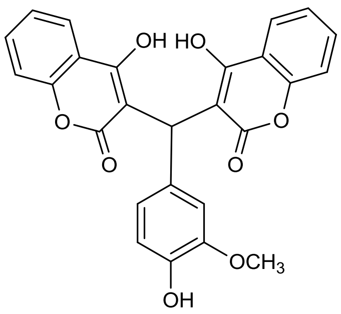

14

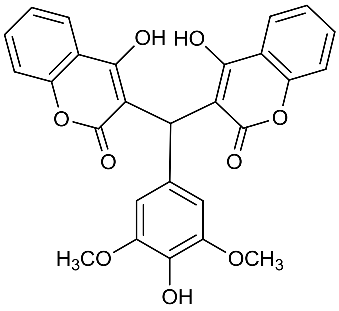

15

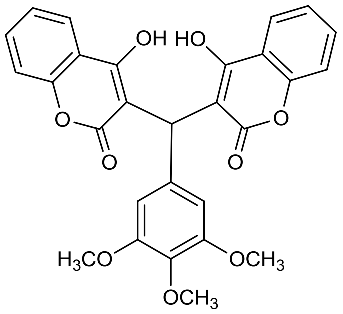

16

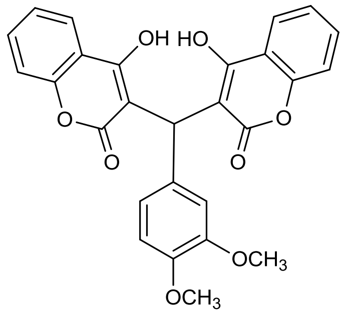

17

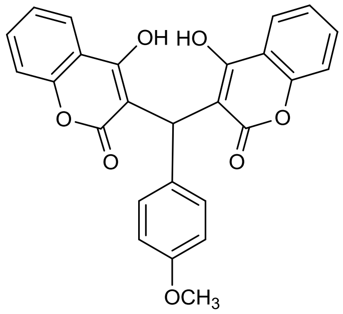

18

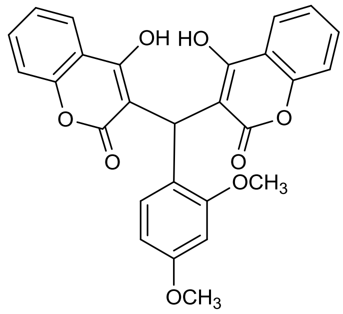

19

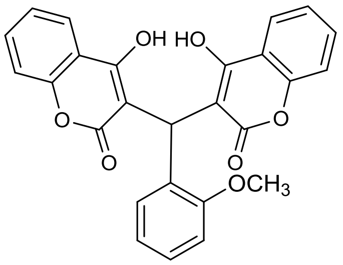

20

Figure S2 Schematic representation of the synthesis compounds 1-20.

Characteristic spectroscopic data

1. 3,3'methylene)bis-(4-hydroxy-2H-chromen-2-one)

Solid; Molecular formula: $C_{19}H_{12}O_6$, Yield 85%; ^{1}H-NMR($CDCl_3$) δ (ppm): 3.85(2H,s), 7.33-7.40(4H,m), 7.59(2H,t), 7.99(2H,d) and 11.30(2H,s)

2. 3,3'-(2,4-dichlorophenylmethylene)bis-(4-hydroxy-2H-chromen-2-one)

Solid; Molecular formula: $C_{25}H_{14}Cl_2O_6$, Yield 72%; ^{1}H-NMR(CDCl3) δ (ppm): 6.02(1H,s), 7.28(2H,d), 7.35-7.40(5H,m), 7.58(2H, t), 8.05(2H,dd), 10.95(1H,s) and 11.72(1H,s)

3. 3,3'-(3,4-dichlorophenylmethylene)bis-(4-hydroxy-2H-chromen-2-one)

Solid; Molecular formula: $C_{25}H_{14}Cl_2O_6$, Yield 76%; ^{1}H-NMR(CDCl3) δ (ppm): 6.01(1H,s), 7.06(1H,d), 7.27(2H,d), 7.36-7.46(4H,m), 7.65(2H, t), 8.04(2H,dd), 11.31(1H,s) and 11.58(1H,s)

4. 3,3'-(2-chlorophenylmethylene)bis-(4-hydroxy-2H-chromen-2-one)

Solid; Molecular formula: $C_{25}H_{15}ClO_6$, Yield 78%; ^{1}H-NMR($CDCl_3$) δ (ppm): 6.14(1H,s), 7.23(3H,m), 7.39(4H,m), 7.43(1H,d), 7.61(2H, t), 8.02(2H,d),10.93(1H,s) 11.63(1H,s)

5. 3,3'-(3-chlorophenylmethylene)bis-(4-hydroxy-2H-chromen-2-one)

Solid; Molecular formula: $C_{25}H_{15}ClO_6$, Yield 80%; ^{1}H-NMR($CDCl_3$) δ (ppm): 6.03(1H,s), 7.24(4H,m), 7.37-7.41(4H,m), 7.62(2H,dt), 8.02(2H,s), 11.29(1H,s) and 11.60(1H,s)

6. 3,3'-(4-chlorophenylmethylene)bis-(4-hydroxy-2H-chromen-2-one)

Solid; Molecular formula: $C_{25}H_{15}ClO_6$, Yield 78%; ^{1}H-NMR($CDCl_3$) δ (ppm): 6.04(1H,s), 7.16(2H,d), 7.28(2H,d), 7.41(4H,m), 7.63(2H, t), 8.04(2H,dd), 11.33(1H,s) and 11.55(1H,s)

7. 3,3'-(4-bromophenylmethylene)bis-(4-hydroxy-2H-chromen-2-one)

Solid; Molecular formula: $C_{25}H_{15}BrO_6$, Yield 75%; ^{1}H-NMR($CDCl_3$) δ (ppm): 6.01(1H,s), 7.10(2H,d), 7.41(6H,m), 7.63(2H, t), 8.02(2H,dd), 11.33(1H,s) and 11.58(1H,s)

8. 3,3'-(phenylmethylene)bis-(4-hydroxy-2H-chromen-2-one)

Solid; Molecular formula: $C_{25}H_{16}O_6$, Yield 71%; ^{1}H-NMR($CDCl_3$) δ (ppm): 6.13(1H,s), 7.20–7.45 (9H,m), 7.61 (2H,t), 8.1 (2H,d) 11.30(1H,s) and 11.60(1H,s)

9. 3,3'-(4-methylphenylmethylene)bis-(4-hydroxy-2H-chromen-2-one)

Solid; Molecular formula: $C_{26}H_{18}O_6$, Yield 82%; ^{1}H-NMR($CDCl_3$) δ (ppm): 2.37(3H,s), 6.04(1H,s), 7.18(4H,t), 7.40(4H,t), 7.61(2H,t), 8.02(2H,dd), 11.28(1H,s) and 11.52(1H,s)

10. 3,3'-(4-tertbutylphenylmethylene)bis-(4-hydroxy-2H-chromen-2-one)

Solid; Molecular formula: $C_{29}H_{24}O_6$, Yield 85%; ^{1}H-NMR($CDCl_3$) δ (ppm): 1.29(9H,s), 6.05(1H,s), 7.14(2H,d), 7.33(2H,d), 7.40(4H,d), 7.62(2H,dt), 8.02(2H,s), 11.28(1H,s) and 11.50(1H,s)

11. 3,3'-(4-hyroxyphenylmethylene)bis-(4-hydroxy-2H-chromen-2-one)

Solid; Molecular formula: $C_{25}H_{16}O_7$, Yield 74%; ^{1}H-NMR($CDCl_3$) δ (ppm): 5.48(1H,s,br), 6.12 (1H,s), 6.79 (2H,d), 7.08(2H,d),7.40 (4H,m), 7.62(2H,t), 8.02(2H,d), 11.31(1H,s) and 11.49(1H,s)

12. 3,3'-(3-nitrophenylmethylene)bis-(4-hydroxy-2H-chromen-2-one)

Solid; Molecular formula: $C_{25}H_{15}NO_8$, Yield 87%; ^{1}H-NMR($CDCl_3$) δ (ppm): 6.13 (1H,s), 7.38-7.60(6H,m), 7.67(2H,t), 7.98-8.19(4H,m), 11.39(1H,s) and 11.58(1H,s)

13. 3,3'-(3-hydroxyphenylmethylene)bis-(4-hydroxy-2H-chromen-2-one)

Solid; Molecular formula: $C_{25}H_{16}O_7$, Yield 84%; ^{1}H-NMR($CDCl_3$) δ (ppm): 6.10(1H,s), 6.70–6.85 (3H,m), 7.22 (1H,t), 7.41(4H,m),7.66 (2H,m) 8.05(2H,d), 11.28(1H,s) and 11.60(1H,s)

14. 3,3'-(4-hydroxy-3-methoxyphenylmethylene)bis-(4-hydroxy-2H-chromen-2-one)

Solid; Molecular formula: $C_{26}H_{18}O_8$, Yield83%; ^{1}H-NMR($CDCl_3$) δ (ppm): 3.74(3H,s), 5.58(1H,s), 6.02(1H,s), 6.66(2H,t), 6.72(1H,d), 6.86(1H,d), 7.40(4H,d),7.61(2H,t), 8.03(1H,d), 11.25(1H,s) and 11.51(1H,s)

15. 3,3'-(4-hyroxy-3,5dimethoxyphenylmethylene)bis-(4-hydroxy-2H-chromen-2-one)

Solid; Molecular formula: $C_{27}H_{20}O_9$, Yield 87%; ^{1}H-NMR($CDCl_3$) δ (ppm): 3.70(6H,s), 6.08(1H,s), 6.42(2H,s), 7.41(4H,d), 7.63(2H,t), 8.02(2H,d), 11.28(1H,s) and 11.53(1H,s)

16. 3,3'-(3,4,5-trimethoxyphenylmethylene)bis-(4-hydroxy-2H-chromen-2-one)

Solid; Molecular formula: $C_{28}H_{22}O_9$, Yield 88%; ^{1}H-NMR($CDCl_3$) δ (ppm): 3.70(6H,s), 3.83(3H,s), 6.06(1H,s), 6.40(2H,s), 7.35-7.42(4H,m), 7.62(2H,t), 8.03(2H,s), 11.28(1H,s) and 11.53(1H,s)

17. 3,3'-(3,4-dimethoxyphenylmethylene)bis-(4-hydroxy-2H-chromen-2-one)

Solid; Molecular formula: $C_{27}H_{20}O_8$, Yield 83%; ^{1}H-NMR($CDCl_3$) δ (ppm): 3.72(3H,s), 3.86(3H,s) 6.08(1H,s), 6.70(1H,s), 6.79(2H,dd), 7.40(4H,d), 7.61(2H,t), 8.03(2H,d) 11.29(1H,s) and 11.53(1H,s)

18. 3,3'-(4-methoxyphenylmethylene)bis-(4-hydroxy-2H-chromen-2-one)

Solid; Molecular formula: $C_{26}H_{18}O_7$, Yield 81%; ^{1}H-NMR($CDCl_3$) δ (ppm): 3.80(3H,s), 6.08(1H,s), 6.87(2H,d), 7.14(2H,d),7.42(4H,d),7.63(2H,t), 8.08(2H,d), 11.30(1H,s) and 11.51(1H,s)

19. 3,3'-(2-methoxyphenylmethylene)bis-(4-hydroxy-2H-chromen-2-one)

Solid; Molecular formula: $C_{26}H_{18}O_7$, Yield 81%; ^{1}H-NMR($CDCl_3$) δ (ppm): 3.58(3H,s), 6.08(1H,s), 6.86(1H,d), 6.95(1H, t),7.24-7.41(6H,m),7.59(2H,t), 8.01(2H,d), 11.18(1H,s) and 11.23(1H,s)

20. 3,3'-(2,4-dimethoxyphenylmethylene)bis-(4-hydroxy-2H-chromen-2-one)

Solid; Molecular formula: $C_{27}H_{20}O_8$, Yield 89%; ^{1}H-NMR($CDCl_3$) δ (ppm): 3.55(3H,s), 3.80(3H,s) 6.01(1H,s), 6.43(2H,m), 7.17 (1H,d), 7.38 (4H,m),7.59(2H,t), 8.02(2H,d), 11.15(1H,s) and 11.18(1H,s)
